# Oral Lichen Planus and Systemic Diseases

**DOI:** 10.1177/00220345251385966

**Published:** 2025-11-18

**Authors:** S. Warnakulasuriya, P. Ramos-García, M.Á. González-Moles

**Affiliations:** 1 Faculty of Dental, Oral & Craniofacial Sciences, King’s College London, London, UK; 2 WHO Collaborating Centre for Oral Cancer, London, UK; 3 School of Dentistry, University of Granada, Granada, Spain; 4 Instituto de Investigación Biosanitaria ibs. GRANADA, Granada, Spain

**Keywords:** autoimmunity, diabetes, oral lichen planus, oral-systemic disease(s), oncology, evidence-based dentistry/health care

## Abstract

The mouth is referred to as “the mirror of health and disease in the body.” This review critically examines the comorbidity between systemic diseases and oral lichen planus, an autoimmune disorder affecting the oral mucosa with malignant potential and of high worldwide prevalence. Research has indicated that patients with oral lichen planus are significantly predisposed to diabetes mellitus (pooled proportion [PP] = 9.77%, odds ratio [OR] = 1.64, *P* < 0.001), Hashimoto thyroiditis (PP = 8.60%, OR = 2.2, *P* < 0.001), hypothyroidism (PP = 8.14%, OR = 1.65, *P* = 0.02), hyperthyroidism (PP = 2.84%, OR = 2.11, *P* = 0.007), celiac disease (PP = 7.14%, OR = 4.09, *P* < 0.001), hepatitis C (PP = 7.14%, OR = 4.09, *P* < 0.001), hepatitis B (PP = 3.90%, OR = 1.62, *P* = 0.02), steatohepatitis (PP = 7.06%, OR = 5.71, *P* = 0.05), liver cirrhosis (PP = 4.27%, OR = 5.8, *P* = 0.002), depression (PP = 31.19%, OR = 6.15, *P* < 0.001), anxiety (PP = 54.76%, OR = 3.51, *P* < 0.001), and stress (PP = 41.10%, OR = 3.64, *P* = 0.005). A good knowledge of these associations may assist primary care physicians, dentists, and other oral health professionals involved in the management of patients with oral lichen planus since many patients may be unaware of these associations and could have an impact on their general health. Some of these diseases, such as diabetes, have a role in the development of oral lichen planus. In addition, most of these comorbidities act as risk factors for cancer of different locations: liver, thyroid, small intestine, and the oral cavity. Current evidence indicates a high prevalence and a higher risk of systemic diseases in patients with oral lichen planus compared with the general population. Future research is recommended to increase our knowledge of pathobiology and clinical management of these associations.

## Introduction

**Figure 1.**
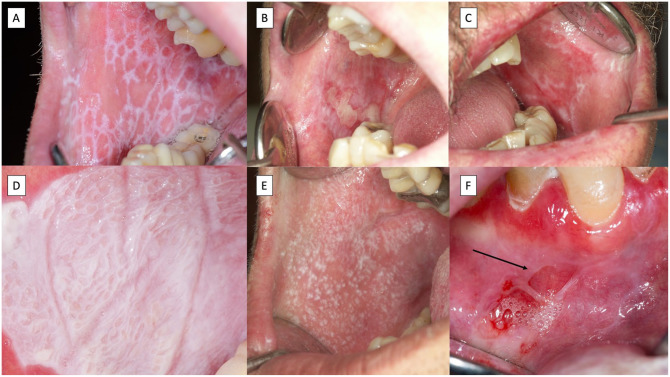
Images corresponding to typical oral lichen planus lesions. (**A**) Reticular lesions. (**B**) Atrophic-erosive reticular lesions. (**C**) Reticular and atrophic lesions. (**D**) Plaque lesions with a reticular picture over the hyperkeratotic plaque. (**E**) Multiple hyperkeratotic papules. (**F**) Desquamative gingivitis in a patient also presenting with blistering lesions with a ruptured blister roof (black arrow).

**Figure 2.**
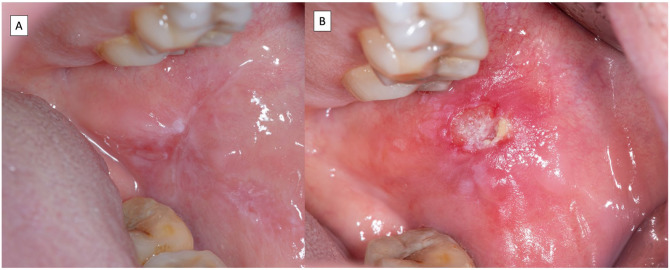
Images corresponding to a patient with erosive, atrophic, and reticular oral lichen planus (**A**) who developed an oral squamous cell carcinoma during the evolution of the disease (**B**).

**Figure 3.**
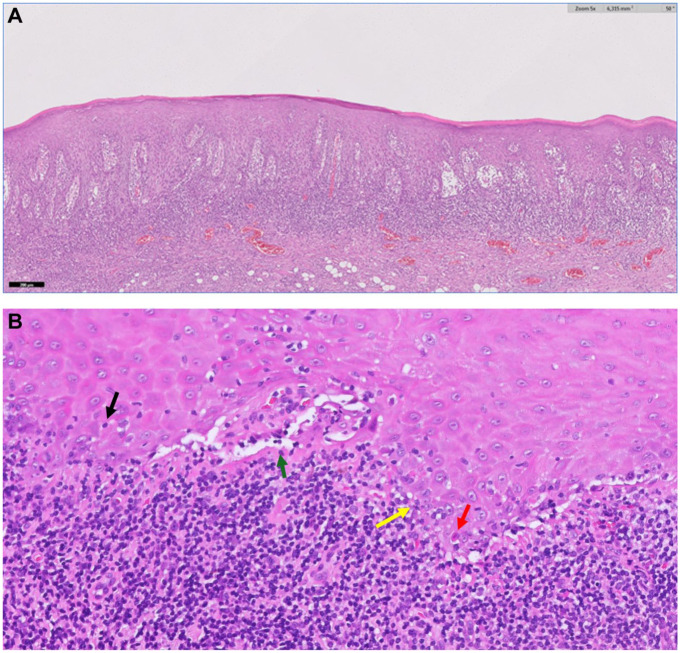
(**A**) Histological image of an oral mucosal biopsy demonstrating the essential histopathological features of the disease: bandlike inflammatory infiltrate in the superficial corium and vacuolating degeneration of the basal layer of the epithelium (hematoxylin and eosin ×20). (**B**) This high-power image illustrates oral mucosal epithelium affected by lichen planus. An intense inflammatory infiltrate with lymphocytes can be observed. Some lymphocytes penetrate the oral epithelium (exocytosis) (black arrow). The immune response causes an alteration in the morphology of the basal epithelial cells and parabasal cells of the oral epithelium (vacuolating or liquefactive degeneration) (yellow arrow), which, when extreme, leads to the appearance of artifactual cleft formation between the epithelium and submucosa (Max Joseph spaces) (green arrow). Some epithelial cells develop apoptotic changes that manifest as a strongly eosinophilic appearance (colloid bodies or Civatte bodies) (red arrow) (hematoxylin and eosin ×200).

Lichen planus is a mucocutaneous disease with a primary involvement of the oral mucosa (González-Moles and Ramos-García 2023). Clinicians should be aware that lichen planus may co-manifest orally and at other mucosal and nonmucosal body sites (e.g., genitals, esophagus, skin, nose, etc.) (Le Cleach and Chosidow 2012). Oral lichen planus (OLP) affects 1% of the general population (González-Moles et al. 2021), and is classified under oral potentially malignant disorders with a malignant transformation ranging from 1.14% to 2.28% (González-Moles, Ramos-García, et al. 2021). The diagnosis of OLP is by clinical examination—the presence of white reticular lesions is a mandatory requirement for the diagnosis of OLP (Figs. 1 and 2)—and pathology should demonstrate vacuolizing degeneration of the basal layer in the oral epithelium, apoptotic keratinocytes, and an inflammatory infiltrate mostly packed with lymphocytes in a bandlike arrangement in the superficial chorion (Lodolo et al. 2023) (Fig. 3A,B).

**Figure 4.**
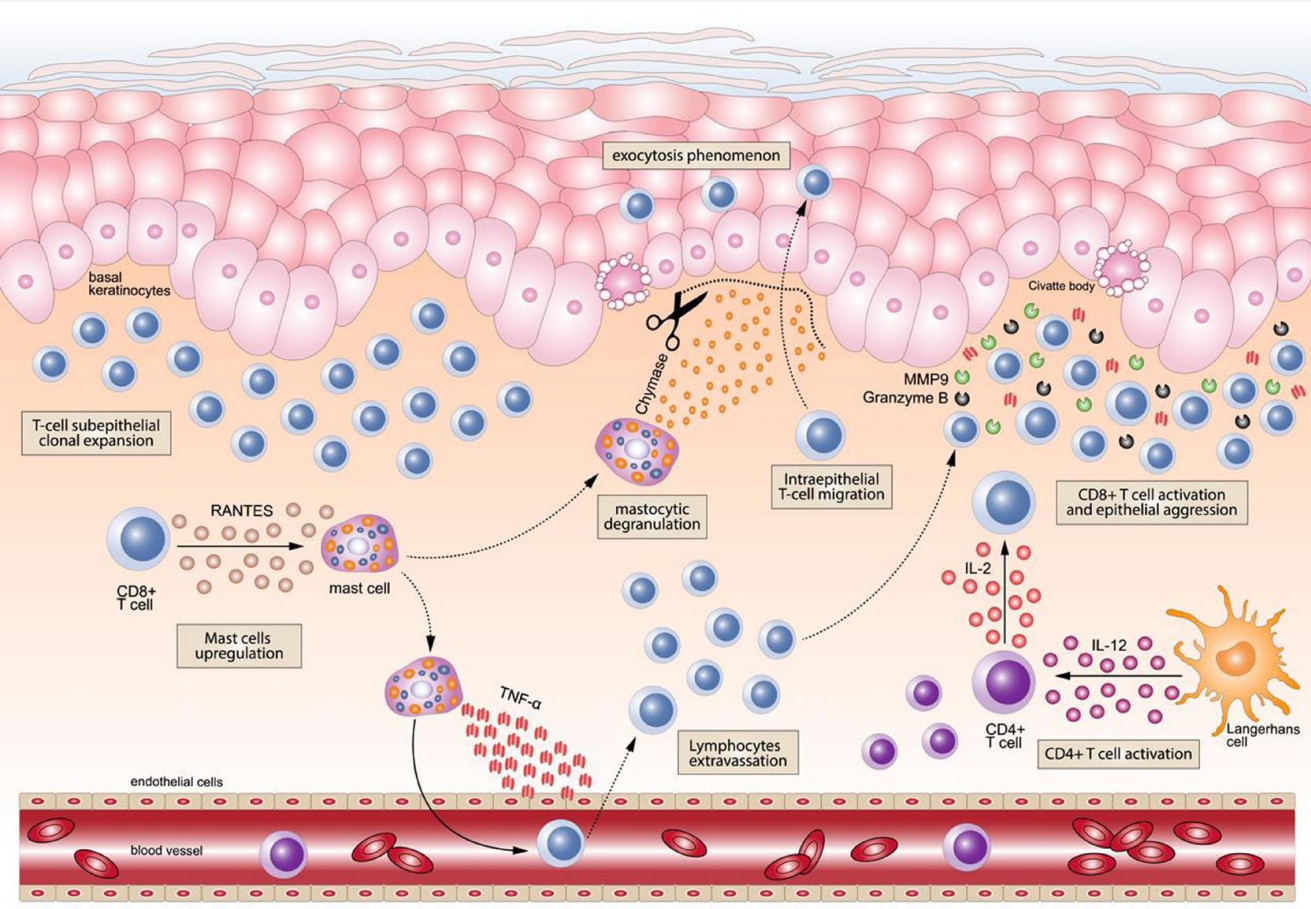
In oral lichen planus etiopathogenesis, cytotoxic T lymphocytes (CD8+) undergo subepithelial clonal expansion at the intralesional inflammatory site. In turn, these lymphocytes may overexpress RANTES and other cytokines, resulting in mast cell upregulation. Among their different roles, mast cells release tumor necrosis factor–α (TNF-α) to mediate the extravasation of T lymphocytes from the blood vessels toward the inflammatory lesional site. This step also increases the amount of T lymphocytes accumulated in the superficial dermis with a bandlike appearance. On the other hand, the upregulated mast cells can also migrate to the subepithelial lymphocyte infiltrate site (the migration of the mast cell is graphically represented by an intermittent line), where they experience a process of intralesional degranulation and the release of the protease chymase, which triggers the degradation of the basement membrane (represented by scissors for illustrative purposes), resulting in a key event for the development of the phenomenon of lymphocyte intraepithelial migration (exocytosis). The intraepithelial location of CD8+ T cells enables the development of cytotoxicity not only on keratinocytes in the basal layer but also on keratinocytes located in the thickness of the epithelium (the intraepithelial CD8+ T-cell migration is graphically represented by the dotted line). Simultaneously, antigen presenting cells (i.e., Langerhans cells) activate CD4+ T cells through the overexpression of cytokines, such as interleukin-12 (IL-12), among others. This in turn promotes the activation of CD8+ T cells through a cytokine-mediated molecular signaling pathway by CD4+ T cells. In summary, all of these mechanisms promote and culminate in the cytotoxic aggression of CD8+ T cells, through the secretion of molecules such as TNF-α, granzyme-B, or matrix metalloproteinase–9 (MMP-9), targeted to the basal and parabasal layers of the epithelium, leading to the development of apoptotic phenomena in keratinocytes (apoptotic or Civatte bodies).

The immunologic events involved in OLP (Fig. 4) begin with the recognition of antigens expressed by basal keratinocytes by CD8+ T lymphocytes and their subsequent activation, which finally generates cytotoxicity mediated by these cells directed toward the basal keratinocytes that develop notable morphological alterations (vacuolizing degeneration) and in some cases apoptosis (Civatte bodies) (El-Howati et al. 2023). The nature of the antigen is usually unknown, although hepatitis C virus (Georgescu et al. 2019; Nagao and Tsuji 2021) as well as autoantigens generating immune cross-reactions, have been implicated, such as Hashimoto’s thyroiditis or hypothyroidism (Robledo-Sierra et al. 2018; Zhang et al. 2022). Most subepithelial and intraepithelial lymphocytes in OLP lesions are CD8+ T cells (DeAngelis et al. 2019; Vičić et al. 2023). CD8+ T lymphocytes could recognize antigens as a consequence of routine surveillance in the epithelium or secondary to the attraction of keratinocyte-derived chemokines (DeAngelis et al. 2019). The cytotoxicity reactions developed in OLP also require the establishment of other molecular mechanisms involving CD4+ T lymphocytes (DeAngelis et al. 2019; Vičić et al. 2023). Basal keratinocyte apoptosis is triggered by cytotoxic signals induced by the proinflammatory signaling pathway (tumor necrosis factor [TNF]–α, granzyme-B, Fas-L, etc.), while mast cell degranulation is induced by T-cell–derived RANTES, which releases TNF-α and chymase, promoting lymphocyte infiltration and basal membrane damage, thus perpetuating the autoimmune response (DeAngelis et al. 2019; Vičić et al. 2023) (Fig. 4).

Recent evidence has shown that OLP patients may present with a significant increase in the risk of developing systemic diseases (Table 1), some of which significantly impair the patient’s general health and quality of life and may also have a direct effect on oral health, behaving in many cases as chronic diseases. Suffering from these comorbidities may also increase the risk of developing different types of cancer, including oral cancer. Often, these comorbidities are unknown to the patient and consequently have not been investigated or managed prior to seeing a dentist or an oral physician. Furthermore, limitations and inconsistencies of published research are critically examined, particularly in the context of the current lack of knowledge about the biological mechanisms underlying the association between OLP and systemic diseases. The aim of this critical review is to present the current knowledge based on evidence on the relationships between OLP and systemic diseases. This review includes the association of OLP with several selected systemic diseases, excluding those with insufficient current evidence, for example, Sjögren’s syndrome.

**Table 1.** Systemic Diseases and OLP.

Diseases in OLP	Prevalence (95% CI)	Magnitude of Association (95% CI), *P* Value	Reference
Depression	31.19% (22.27–40.82)	OR = 6.15 (2.72–13.89), *P* < 0.001	De Porras-Carrique et al. (2022)
Anxiety	54.76% (42.06–67.17)	OR = 3.51 (2.10–5.85), *P* < 0.001	De Porras-Carrique et al. (2022)
Stress	41.10% (32.18–50.32)	OR = 3.64 (1.48–8.94), *P* = 0.005	De Porras-Carrique et al. (2022)
Diabetes mellitus	9.41% (8.16–10.74)	OR = 1.64 (1.34–2.00), *P* < 0.001	De Porras-Carrique et al. (2023)
Hypertension	24.17% (21.45–27.00)	OR = 1.28 (1.01–1.63), *P* = 0.04	De Porras-Carrique et al. (2024)
Fibromyalgia	3.95% (0.07–11.17)	OR = 1.74 (0.06–50.43), *P* = 0.75	De Porras-Carrique et al. (2023)
Inflammatory bowel disease	5.01% (1.07–10.85)	OR = 5.08 (0.24–106.80), *P* = 0.30	De Porras-Carrique et al. (2023)
Celiac disease	8.66% (4.71–13.43)	OR = 18.44 (2.40–141.43), *P* = 0.005	De Porras-Carrique et al. (2023)
Rheumatoid arthritis	2.40% (0.77–4.65)	OR = 2.34 (0.34–16.13), *P* = 0.34	De Porras-Carrique et al. (2023)
Sjögren’s syndrome	2.96% (0.77–6.14)	OR = 4.41 (0.68–28.43), *P* = 0.12	De Porras-Carrique et al. (2023)
Lupus erythematosus	1.65% (0.10–4.35)	OR = 3.02 (0.12–74.90), *P* = 0.50	De Porras-Carrique et al. (2023)
Psoriasis	0.82% (0.04–2.20)	OR = 0.50 (0.04–5.54), *P* = 0.57	De Porras-Carrique et al. (2023)
Vitiligo	1.83% (0.02–5.33)	OR = 3.05 (0.12–76.26), *P* = 0.50	De Porras-Carrique et al. (2023)
Hashimoto’s thyroiditis	8.60% (3.78–14.94)	OR = 2.23 (1.76–2.82), *P* < 0.001	De Porras-Carrique et al. (2023)
	NR	OR = 2.29 (1.43–3.67), *P* < 0.001	Serni et al. (2024)
Hyperthyroidism	2.84% (1.31–4.87)	OR = 2.11 (1.22–3.63), *P* = 0.007	De Porras-Carrique et al. (2023)
Hypothyroidism	8.14% (6.07–10.45)	OR = 1.65 (1.08–2.53), *P* = 0.02	De Porras-Carrique et al. (2023)
Hepatitis B	3.90% (1.90–6.44)	OR = 1.62 (1.01–2.40), *P* = 0.02	González-Moles et al. (2023)
Hepatitis C	7.14% (5.46–9.00)	OR = 4.09 (2.77–6.03), *P* < 0.001	González-Moles et al. (2023)
	9.42% (7.27–11.58)	OR = 4.48 (3.48–5.77), *P* = nr	García-Pola et al. (2023)
Steatohepatitis	7.06% (1.51–15.47)	OR = 5.71 (0.97–33.60), *P* = 0.05	González-Moles et al. (2023)
Liver cirrhosis	4.27% (1.22–8.58)	OR = 5.58 (1.83–16.96), *P* = 0.002	González-Moles et al. (2023)
Hepatocellular carcinoma	9.13% (4.12–15.66)	OR = 0.94 (0.37–2.40), *P* = 0.90	González-Moles et al. (2023)

Abbreviations: CI, confidence intervals; OLP, oral lichen planus; OR, odds ratio; NR, not reported.

## OLP and Diabetes Mellitus

Contradictory results were found when analyzing the relationship between lichen planus and diabetes mellitus (Otero Rey et al. 2019). A recent meta-analysis on 116 studies and 17,609 patients (De Porras-Carrique et al. 2023) has shown that 9.77% of patients with OLP are diabetic and also that OLP patients have a significantly higher risk of suffering from DM as compared with the general population (odds ratio [OR] = 1.64, *P* < 0.001). The data reported mainly derives from type 2 diabetes mellitus (DM). A potential limitation across primary studies is the lack of specific information on type 1 DM, reported in only 5 primary studies/489 patients, which showed a prevalence of 1.62% in OLP patients (De Porras-Carrique et al. 2023). A published indirect estimate (González-Moles, Warnakulasuriya, et al. 2021; De Porras-Carrique et al. 2023) indicates that 7.8 million patients with OLP in the world are diabetic and that 2.8 million of these patients with OLP are unaware that they suffer from diabetes (Beagley et al. 2014; Ogurtsova et al. 2017).

The underlying biological mechanisms of the association between OLP and DM have not yet been fully elucidated. OLP shares with some forms of diabetes—type 1 and latent autoimmune diabetes in adults—an etiopathogenesis of autoimmune origin. It is well known that these diabetic patients frequently present a “state of systemic autoimmunity” (Miller 2023), with a predisposition to develop multiple autoimmune diseases simultaneously, the most frequent in diabetic patients being Hashimoto’s thyroiditis (Gougourelas et al. 2021). Patients with OLP and type 2 DM have a common background of a chronic systemic inflammatory state, characterized by elevated levels of cytokines such as TNF-α, interleukin (IL)–6, and IL-1β (Rohm et al. 2022; Vičić et al. 2023). This shared inflammatory environment may play a role in the immunopathogenesis of both diseases. It could promote the activation of autoreactive T lymphocytes targeting basal keratinocyte antigens in OLP. While this association remains to be conclusively demonstrated, the overlap between autoimmunity and systemic inflammation suggests a possible mechanistic link worthy of further investigation. Current evidence suggests that the association between DM and OLP could be bidirectional (Yao et al. 2024). Chronic systemic inflammation and, in some cases, autoimmunity may predispose diabetic patients to develop or exacerbate OLP lesions (Rohm et al. 2022; Vičić et al. 2023; Yao et al. 2024), while topical or systemic corticosteroids used to treat OLP may elevate blood glucose levels, representing an additional consideration in diabetic patients (Golubic et al. 2025). Clinically, this relationship highlights the importance of interdisciplinary management to optimize glycemic control in patients with diabetes and OLP. In patients with diabetes receiving corticosteroid therapy for OLP, glucose monitoring should be implemented to prevent hyperglycemia-related complications.

## OLP and Hypertension

A syndromic association between OLP, DM, and hypertension was reported by Grinspan in 1966 (Garlapati et al. 2017). It has been demonstrated on the basis of the evidence (104 primary level studies/16,587 cases) that OLP patients also develop hypertension in 24.2% of cases (95% confidence interval [CI] = 21.45–27.00) with the risk of hypertension being 1.28 times higher than in the general population (95% CI = 1.01–1.63, *P* = 0.05) (De Porras-Carrique et al. 2024). However, due to limitations noted in these studies, the evidence for this association is considered weak and, in any case, subject to further confirmation. There is no meta-analysis that provides information on the prevalence and risk of concomitant hypertension and DM in the population of OLP patients; thus, Grisnpan’s syndrome is currently in question (Garlapati et al. 2017).

Certain evidence justifies the biological plausibility of the mechanisms involved in the OLP and hypertension association. Patients with OLP have chronic immune activation, which, as discussed above, may generate a systemic proinflammatory state (Vičić et al. 2023) characterized by the release of cytokines and chemokines. These inflammatory molecules are thought to maintain high circulating levels and may perhaps be involved in endothelial dysfunction and peripheral vascular resistance, key mechanisms in the pathogenesis of hypertension (Guzik et al. 2024). Other potentially implicated mechanisms are emotional disturbances (depression, anxiety, and stress) that frequently occur in patients with OLP. These states promote the release of catecholamines and cortisol, which increase heart rate and peripheral vasoconstriction, leading to hyperactivity of the sympathetic nervous system, being well-established factors in the underlying cause of hypertension (Levine et al. 2021). The association between OLP and hypertension appears to be unidirectional, with OLP potentially predisposing to or exacerbating high blood pressure rather than the reverse. This relationship may be mediated by systemic inflammation and chronic immune activation in OLP, which may contribute to endothelial dysfunction and vascular changes involved in hypertension (Vičić et al. 2023; Guzik et al. 2024). Moreover, corticosteroids, commonly used in the management of OLP, can further elevate blood pressure (Mebrahtu et al. 2020). These observations underline the need for clinicians to regularly monitor blood pressure in patients with OLP, particularly when corticosteroid therapy is prescribed.

## OLP and Thyroid Disease

Patients with OLP have a significantly higher risk of developing thyroid disease than the general population does. The available evidence derived from a meta-analysis on 75 primary level studies and 15,176 OLP patients provides information on Hashimoto’s thyroiditis, hypothyroidism, and hyperthyroidism (De Porras-Carrique et al. 2023; Serni et al. 2024).

Hashimoto’s thyroiditis is the most prevalent thyroid pathology in patients with OLP (8.60%), with the risk of developing it being 2.2 times higher than in the general population (*P* < 0.001) (De Porras-Carrique et al. 2023; Serni et al. 2024). It is an autoimmune disorder that progressively destroys the thyroid gland, leading to hypothyroidism (Chaker et al. 2022), or it can remain asymptomatic (Ralli et al. 2020). Patients with Hashimoto’s thyroiditis have a significant risk of developing some types of cancers, probably linked to the establishment of a chronic autoimmune phenomenon; among the most frequent are thyroid, colon, breast, uterus, prostate cancers, and thyroid lymphomas (Chen et al. 2013). There is no evidence on the risk of oral cancer in patients with OLP and Hashimoto’s thyroiditis, an aspect that should be investigated in the future.

Hypothyroidism is the second most prevalent thyroid pathology in patients with OLP (8.14% of cases), with a risk of developing the disease 1.65 times higher than in the general population (*P* = 0.02) (De Porras-Carrique et al. 2023). However, the symptoms of hypothyroidism can develop insidiously, and it is likely that some patients with OLP are unaware that they suffer also from hypothyroidism; consequently, the dentist should be aware of the most common symptoms of hypothyroidism (fatigue, weight gain, bradycardia and hoarseness, among others) in order to suspect it in their patients with OLP who present to them. In some cases, hypothyroidism may also be asymptomatic (subclinical hypothyroidism) (Chaker et al. 2022).

Hyperthyroidism develops in 2.84% of patients with OLP, with the frequency 2.11 times higher in OLP compared with the general population (*P* = 0.007) (De Porras-Carrique et al. 2023). The most common cause of hyperthyroidism in the general population is Graves–Basedow disease (Davies et al. 2020), an autoimmune disorder, and this disease is possibly also a frequent cause of hyperthyroidism in patients with OLP, although there is not enough evidence for this. The most common symptoms of hyperthyroidism are weight loss, muscle weakness, tremor, tachycardia, atrial fibrillation, neuropsychiatric disorders, and insomnia, among others (Davies et al. 2020).

The association between Hashimoto’s thyroiditis/hypothyroidism and OLP could reflect a phenomenon of cross-immunity, in which the immune system may target shared or structurally similar antigens in the thyroid gland and oral mucosa. Both diseases may be linked to an autoimmune substrate mediated by cytotoxic T-lymphocytes (CD8+) and autoantibodies (Guarneri et al. 2017; Zhang et al. 2022). In thyroid diseases, antithyroglobulin and antithyroperoxidase antibodies attack the thyroid gland, whereas in OLP, T lymphocytes infiltrate the oral mucosa, recognizing and insulting unknown epithelial antigens. In this regard, it has been suggested that certain peptides of thyroglobulin or thyroperoxidase may have structural similarities to proteins of the oral epithelium, triggering a cross-immune response (Wu et al. 2020). On the other hand, a mechanistic connection between autoimmune thyroid diseases and OLP has been suggested through the analysis of thyroid-stimulating hormone receptor (TSHR) expression in OLP patients (Robledo-Sierra et al. 2018). It was demonstrated that TSHR is highly expressed in the OLP lesions of patients with thyroid disease compared with healthy oral mucosa, suggesting that TSHR may be involved in the pathogenesis of OLP.

The association between OLP and thyroid diseases has been demonstrated in meta-analytic studies (De Porras-Carrique et al. 2023; Serni et al. 2024), but current evidence does not indicate the direction of causality, whether thyroid dysfunction predisposes to OLP, OLP favors thyroid autoimmunity, or whether both conditions simply coexist due to a shared autoimmune background. Nevertheless, despite the weak evidence, clinicians should remain alert to hypothyroid or hyperthyroid symptoms during OLP management, as undiagnosed thyroid dysfunction may be relatively common.

## OLP and Celiac Disease

Patients with OLP suffer from celiac disease in 8.66% of cases, with a risk 18.44 times higher than general population (*P* = 0.005) (De Porras-Carrique et al. 2023; Martín-Masot et al. 2025). The prevalence of celiac disease in the general population is about 1.4% (King et al. 2020) and, because of its wide and variable symptomatology and the fact that it can be asymptomatic, it frequently goes unnoticed (Lindfors et al. 2019).

Underlying mechanisms have been reported that could justify the OLP–celiac disease association, but evidence is currently weaker than for the other diseases investigated (De Porras-Carrique et al. 2023; Martín-Masot et al. 2025). In this regard, cross-autoimmunity and mimetic molecular phenomena between both diseases have also been hypothesized (Cigic et al. 2015). Tissue transglutaminase, a target enzyme in celiac disease, might have a structural homology with proteins of the oral epithelium, allowing circulating T lymphocytes activated by gluten to recognize similar antigens of the oral mucosa and to participate in the pathogenesis of OLP (Cigic et al. 2015; Martucciello et al. 2020). On the other hand, it has been reported that the immune response to gluten in celiac patients may attack the oral mucosa, causing epithelial disruption (Sanchez-Solares et al. 2021). Epithelial disruption in the oral mucosa of patients with celiac disease has been documented by a significant decrease in the expression of epithelial junction proteins, which compromises the integrity of this mucosal barrier (Sanchez-Solares et al. 2021). This mechanism could also have implications for the pathogenesis of OLP patients. Taken together, the current evidence remains limited and should be interpreted with caution, and no definitive causal direction has been established (De Porras-Carrique et al. 2023; Martín-Masot et al. 2025). Therefore, systematic screening for celiac disease in OLP patients cannot yet be recommended. However, clinicians should remain alert to gastrointestinal or extraintestinal symptoms suggestive of celiac disease.

## OLP and Liver Disorders

Patients with OLP have a significantly higher incidence of liver pathology than in the general population. The available evidence (146 primary-level studies/21,187 OLP patients) offers information on C and B hepatitis, steatohepatitis, and liver cirrhosis (García-Pola et al. 2023; González-Moles et al. 2023; Liu et al. 2024). These liver diseases are risk factors for the development of hepatocellular carcinoma.

**Figure 5.**
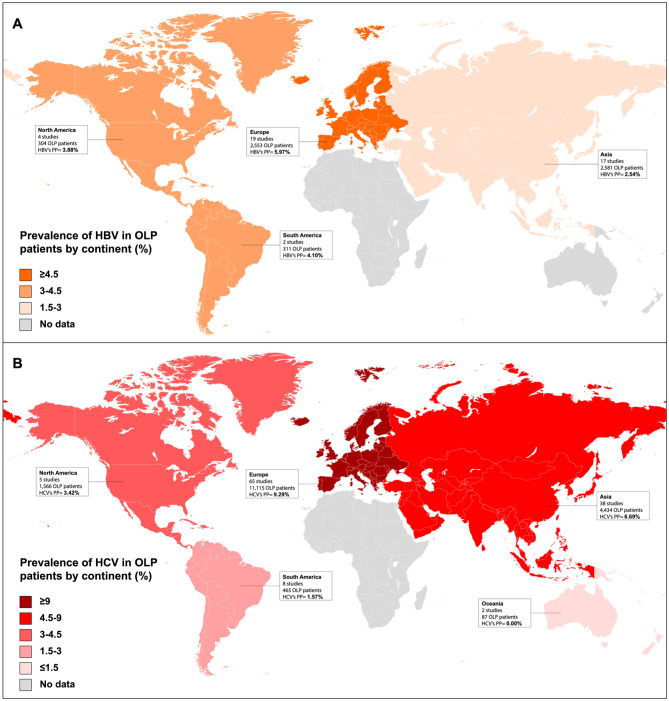
These maps depict the pooled prevalence of hepatitis B and C in oral lichen planus patients stratified by continents. (**A**) The highest prevalence of hepatitis B was found in Europe (pooled proportion [PP] = 5.97%), whereas the lowest was found in Asian countries (PP = 2.54%). Under reporting from South and East Asian Countries is a likely factor for lower prevalence (**B**) Europe also represented the continent with the highest prevalence of hepatitis C (PP = 9.29%), whereas no cases were detected in Oceania (PP = 0.00%).

Patients with OLP suffer concomitantly from hepatitis C in 7.14% of cases, with the risk of developing the disease being 4.09 times higher than in the general population (*P* < 0.001). It has also been shown that the prevalence of hepatitis C in OLP presents geographical differences, being significantly higher in Europe where 9.29% of OLP cases suffer from it (González-Moles et al. 2023) (Fig. 5A). Hepatitis C is the most common liver disease associated with hepatocellular carcinoma in North America, Europe, and Japan (Llovet et al. 2021). It should be noted that the hepatitis C virus is an RNA virus that does not integrate its genome, and consequently, its oncogenicity is not linked to the genesis of insertional mutations but has the capacity to develop liver cirrhosis. This is important because it is estimated that hepatitis C goes unnoticed in 45% to 85% of patients (Manns et al. 2017), some of whom will develop liver cirrhosis. Hepatitis C also increases the risk of oral cancer in patients with OLP (Relative Risk [RR] = 3.67, *P* = 0.005) (González-Moles et al. 2023). The diagnosis of these cases of asymptomatic hepatitis C is important because direct-acting antiviral therapy achieves a sustained virological response in a high percentage of patients and reduces the risk of developing hepatocellular carcinoma by 50% to 80% (Manns et al. 2017). Furthermore, evidence has also shown that patients with hepatitis C have a high prevalence of OLP (9.42% of cases), with an OLP risk 4.48 times higher than in the healthy population (*P* < 0.05) (García-Pola et al. 2023).

Patients with OLP present with hepatitis B in 3.90% of cases, and significant geographical differences in its distribution are observed, with Europe being the most prevalent endemic area (5.97% of European patients with OLP suffer concomitantly from hepatitis B) (Fig. 5B). The risk of developing hepatitis B in OLP is 1.62 times higher than in the general population (*P* = 0.02) (González-Moles et al. 2023). This association is relevant because it is known that, as with hepatitis C, a number of hepatitis B patients are asymptomatic (Llovet et al. 2021) and 25% of patients with chronic, untreated hepatitis B die from liver cirrhosis or hepatocellular carcinoma (Llovet et al. 2021).

Patients with OLP have a high prevalence of steatohepatitis (7.06%), with a risk of developing the disease 5.71 times higher than in the general population (*P* = 0.05) (González-Moles et al. 2023). Steatohepatitis is a known risk factor for liver cirrhosis and for hepatocellular carcinoma. Nonalcoholic steatohepatitis is the most common cause of liver cirrhosis in many areas of the world, and it is currently recognized that 15% to 20% of hepatocellular carcinomas in Western countries derive from steatohepatitis (Estes et al. 2018). Steatohepatitis is by nature an asymptomatic process, so OLP patients are often unaware of their condition (Sheka et al. 2020).

Finally, patients with OLP also have a high prevalence of liver cirrhosis (4.27%), with a risk of developing the disease 5.8 times higher than the general population (*P* = 0.002) (González-Moles et al. 2023), which probably derives from the high prevalence of precancerous liver diseases in patients with OLP (C and B hepatitis and steatohepatitis). The tissue and molecular disorders that occur in liver cirrhosis transform the liver into a cancerous field with a high risk of developing hepatocellular carcinoma (Llovet et al. 2021). Today it is known that 90% of liver carcinomas derive from the evolution of cirrhosis (Sheka et al. 2020).

With regard to the cause of this association, it is well known that the immune aggression developed in OLP starts with the recognition of antigens expressed by basal keratinocytes. Hepatitis C virus (HCV) could be a target antigen presented by Langerhans cells, followed by the aggression of T lymphocytes to basal cells (Georgescu et al. 2019). It should also be noted that it is well known that this virus can be present and replicate in extrahepatic tissues (Rosenthal and Cacoub 2015) and that hepatitis C virus RNA has been demonstrated in lesions of lichen planus (Nagao et al. 2000). On the other hand, HCV and HBV infection induce a chronic inflammatory response with production of proinflammatory cytokines, essentially TNF-α, IL-1, and IL-6, which could also be involved in the pathogenesis of OLP (Bader El Din and Farouk 2024). A recent study reported considerable clinical improvement in HCV-infected OLP patients treated with antiviral therapy (Nagao and Tsuji 2021), which reinforces the evidence for this causal relationship. The available evidence points out a unidirectional link from viral hepatitis to OLP pathogenesis, as demonstrated by the presence of viral antigens and HCV RNA within oral mucosal lesions (Nagao et al. 2000), the elevated systemic cytokine levels in patients with HCV-related OLP (Bader El Din and Farouk 2024), and the clinical improvement of OLP following successful antiviral therapy (Nagao and Tsuji 2021). Although B and C hepatitis and steatohepatitis entail a risk of cirrhosis and hepatocellular carcinoma, opportunities for targeted management are limited by the often asymptomatic nature of liver disease. There is currently limited evidence as to whether OLP patients would benefit from liver disease screening.

## OLP and Emotional Disorders

OLP patients have a significantly higher prevalence and risk of developing emotional disorders compared with the general population. The available evidence (derived from 51 primary-level studies and 6,815 OLP patients) offers information on depression, anxiety, and stress (De Porras-Carrique et al. 2022). The prevalence of depression in OLP is 31.2% of cases, with the risk of developing depressive disorders being 6.15 times higher than in the general population (*P* < 0.001). Anxiety symptoms appear in 54.8% of the population with OLP, which has a risk 3.51 times higher than the general population (*P* < 0.001). Finally, patients affected by OLP suffer from emotional stress in 41.1% of cases, with the risk also being significantly higher than that of the control population (OR = 3.64, *P* = 0.005) (De Porras-Carrique et al. 2022).

There is a biological plausibility underlying this association: emotional disorders and chronic stress favor the release of cytokines and promote a systemic proinflammatory state, which could trigger or exacerbate flare-ups in OLP patients (Harsanyi et al. 2022). In patients with OLP and emotional disorders, the abnormal expression of cytokines and chronic stress markers has been found in oral lesions, serum, and saliva (Simoura et al. 2023; Vičić et al. 2023; Khan et al. 2024), Worsening of OLP symptoms during episodes of increased emotional alterations is commonly reported (Chen et al. 2017). Thus, the causal direction appears predominantly unidirectional, with emotional disorders likely acting as upstream contributors, which may trigger or exacerbate OLP lesions (Chen et al. 2017; Harsanyi et al. 2022). Given the plausible association, clinicians managing OLP should remain alert to symptoms of depression, anxiety, or stress among their patients. Early identification and referral to mental health specialists is recommended (De Porras-Carrique et al. 2022), as coordinated management ay help reduce the severity or frequency of OLP flare-ups and improve the overall quality of life of patients (Chen et al. 2017).

## Limitations and Strengths

Potential limitations of the studies should also be addressed. The diagnostic criteria used to define OLP cases are variable across the primary studies included in the meta-analyses. Over the past several decades, multiple diagnostic frameworks have been proposed, including the World Health Organization of 1978 (Kramer et al. 1978), van der Waal criteria (2003–2007) (van der Meij et al. 2007), and, more recently, the position paper of the American Academy of Oral and Maxillofacial Pathology (Cheng et al. 2016). However, the latter were recently published and, as a result, were not adopted by the earlier primary studies included in the comprehensive systematic reviews published on this topic. It is important to note that all primary-level studies considered the presence of reticular white lesions as an essential diagnostic criterion. Most studies performed biopsies to confirm OLP histopathological findings. Some studies excluded cases with dysplasia, a practice that remains controversial given the lack of strong evidence and its implications for malignancy risk assessment. This lack of uniformity in diagnostic criteria reflects a broader issue within the literature, as there also remains significant controversy among experts regarding which criteria should be considered definitive, particularly given that some proposed diagnostic elements lack solid evidence-based validation. Nevertheless, we believe that despite these inconsistencies, the clinical and histological profiles of the patients included across studies are sufficiently reliable to support the diagnosis of OLP in most—or even all—cases. Therefore, the internal validity of associations between OLP and systemic comorbidities is robust, which was the primary focus of our analysis. Another potential limitation is the current lack of evidence-based knowledge directly supporting specific management strategies tailored to these comorbidities. Therefore, another recommendation from this critical review is the need for future primary level studies to explore in greater depth how these systemic conditions may influence the treatment and clinical course of OLP.

As also specifically discussed, disease by disease, multiple underlying biological mechanisms have been reported with biological plausibility that reinforces the current evidence. Nevertheless, this review also recommends that future basic mechanistic studies should focus on better understanding the etiopathogenic phenomena that link OLP and systemic diseases.

The differential value of this article, compared with previous reports (Hasan et al. 2019; Dave et al. 2021), consists in synthesizing recent, high-quality evidence on the prevalence and magnitude of associations between systemic diseases and OLP, with results derived exclusively from meta-analytic studies published in the past 3 y. We have also explored the underlying etiopathogenic mechanisms linking these associations, which, on the basis of biological plausibility, reinforce the current evidence on these associations. Other strengths include that the quality and risk of bias were evaluated by most of the systematic reviews critically analyzed in the current article. Singularly, a specific tool for systematic reviews addressing prevalence questions—designed by the Joanna Briggs Institute (University of Adelaide, Australia) (Munn et al. 2014)—was consistently applied (De Porras-Carrique et al. 2022; De Porras-Carrique et al. 2023; González-Moles et al. 2023; De Porras-Carrique et al. 2024). This tool covered 10 domains potentially biased across observational studies, singularly focused on confounding factors and selection bias (Munn et al. 2014). Future studies should also take into account important variables that could not be analyzed, for example, indications such as whether OLP patients might be more health conscious and therefore more likely to be evaluated and diagnosed with a systemic disorder. All these aspects should be taken into account as perspectives to analyze the casual relationships based on a higher methodological quality and to standardize future research.

## Conclusions

OLP is associated with several comorbidities that must be recognized by dentists, family doctors, and other oral health professionals involved in the management of OLP patients. Current evidence seems to point out a high prevalence and a higher risk of developing DM, thyroid pathologies, celiac disease, liver pathologies, and emotional disorders in patients with OLP compared with the general population. Clinicians should consider the potential impact of these comorbidities on OLP management: monitoring glycemia and arterial pressure, awareness of symptoms suggestive of thyroid dysfunction or celiac disease, vigilance for asymptomatic liver disorders (particularly hepatitis B and C and steatohepatitis), and referral to mental health specialists for emotional disorders. While these measures are suggested based on the currently available evidence, future methodologically well-designed studies are required to clarify causal pathways and to define precise, evidence-based management strategies for patients with OLP and systemic diseases.

## Author Contributions

S. Warnakulasuriya, contributed to conception and design, data acquisition, critically revised the manuscript; Pablo Ramos-García, contributed to design, data acquisition and analysis, drafted and critically revised the manuscript; M.Á. González-Moles, contributed to conception and design, data acquisition, drafted and critically revised the manuscript. All authors gave their final approval and agree to be accountable for all aspects of the work.
